# Hypoxia-Induced Aggressiveness of Pancreatic Cancer Cells Is Due to Increased Expression of VEGF, IL-6 and miR-21, Which Can Be Attenuated by CDF Treatment

**DOI:** 10.1371/journal.pone.0050165

**Published:** 2012-12-13

**Authors:** Bin Bao, Shadan Ali, Aamir Ahmad, Asfar S. Azmi, Yiwei Li, Sanjeev Banerjee, Dejuan Kong, Seema Sethi, Amro Aboukameel, Subhash B. Padhye, Fazlul H. Sarkar

**Affiliations:** 1 Department of Pathology, Karmanos Cancer Institute, Wayne State University, Detroit, Michigan, United States of America; 2 Department of Oncology, Karmanos Cancer Institute, Wayne State University, Detroit, Michigan, United States of America; 3 ISTRA, Department of Chemistry, MCE Society's Abeda Inamdar Senior College of Arts, Science and Commerce, Pune, India; Southern Illinois University School of Medicine, United States of America

## Abstract

Hypoxia is known to play critical roles in cell survival, angiogenesis, tumor invasion, and metastasis. Hypoxia mediated over-expression of hypoxia-inducible factor (HIF) has been shown to be associated with therapeutic resistance, and contributes to poor prognosis of cancer patients. Emerging evidence suggest that hypoxia and HIF pathways contributes to the acquisition of epithelial-to-mesenchymal transition (EMT), maintenance of cancer stem cell (CSC) functions, and also maintains the vicious cycle of inflammation-all which lead to therapeutic resistance. However, the precise molecular mechanism(s) by which hypoxia/HIF drives these events are not fully understood. Here, we show, for the first time, that hypoxia leads to increased expression of VEGF, IL-6, and CSC signature genes Nanog, Oct4 and EZH2 consistent with increased cell migration/invasion and angiogenesis, and the formation of pancreatospheres, concomitant with increased expression of miR-21 and miR-210 in human pancreatic cancer (PC) cells. The treatment of PC cells with CDF, a novel synthetic compound inhibited the production of VEGF and IL-6, and down-regulated the expression of Nanog, Oct4, EZH2 mRNAs, as well as miR-21 and miR-210 under hypoxia. CDF also led to decreased cell migration/invasion, angiogenesis, and formation of pancreatospheres under hypoxia. Moreover, CDF decreased gene expression of miR-21, miR-210, IL-6, HIF-1α, VEGF, and CSC signatures *in vivo* in a mouse orthotopic model of human PC. Collectively, these results suggest that the anti-tumor activity of CDF is in part mediated through deregulation of tumor hypoxic pathways, and thus CDF could become a novel, and effective anti-tumor agent for PC therapy.

## Introduction

Pancreatic cancer (PC) is one of the most deadly malignant diseases with the poorest clinical outcome in the world. In 2012, it has been estimated that 43, 920 subjects will be newly diagnosed with PC, and will account for 37, 390 cancer-related death in the United States [1]. Due to the absence of specific symptoms, the lack of early detection techniques, and highly aggressive phenotypes, PC is usually diagnosed at an advanced-incurable and metastatic stages [2], [3]. Thus, the median overall survival is approximately six months after surgical and chemo-radiation therapies for locally advanced and metastatic stages of PC. Consequently, the five-year overall survival rate is less than five percent. Such a shorter survival rate is primarily due to late diagnosis and therapeutic resistance, contributing to tumor recurrence and metastasis.

Hypoxia is one of the fundamental biological phenomena that are strongly associated with the development and aggressiveness of a wide variety of solid tumors including PC. Hypoxia-inducible factors (HIF) are a central transcription factor that mediates hypoxia responsive genes and have been widely accepted to play critical roles in tumor invasion, metastasis, and treatment resistance, due to its increased cell proliferation, survival, angiogenesis and cell migration and invasion [4], [5]. Therefore, tumor hypoxia with altered expression of HIF and its biological effect result in poorer clinical outcome of patients diagnosed with solid tumors, resulting in higher mortality, which suggests that understanding of the molecular relationship of hypoxia with other molecular and cellular features of tumor aggressiveness, would be invaluable for developing novel therapeutic strategies for the treatment of solid tumor development and progression. It has been widely accepted that cancer stem cells (CSCs) and epithelial-to-mesenchymal transition (EMT) phenotypic cells are highly associated with therapeutic resistance and contributes to aggressive tumor growth, invasion, and metastasis, and are commonly considered to be one of the major causes of tumor recurrence and relapse [6]. The data from increased number of studies indicate that hypoxia and HIF signaling pathway lead to enrichment of CSCs and EMT cells as reviewed recently [7], contributing to tumor aggressive phenotypes, which could also be due to deregulation of microRNAs (miRNAs).

The miRNAs are well recognized to play pivotal roles in a wide array of biological processes, such as cell differentiation, proliferation, death, survival, metabolism and energy homeostasis [8], [9]. Accumulating evidence suggests that miRNAs might have a critical role in the development and progression of malignant disaeses. The alternations of miRNA expression have been reportedly associated with clinical outcome of tumor patients, treatment resistance, tumor recurrence and/or relapse. A great number of miRNAs have been reported to be responsive to hypoxia and HIF signaling pathway in a wide variety of cells and tissues including cancer cells [10]–[13]. It has been found that hypoxia decreased the expression of miR-101, a potential anti-oncogenic molecule, and increased expression of miR-21 and miR-210, pro-oncogenic molecules in several cancers including PC [14], [15]. Thus, hypoxia-mediated regulation of miRNAs may play important roles in tumor aggressive phenotypes mediated through the modulation of cellular signaling pathways including HIF signaling pathway within a tumor microenvironment. Therefore, targeting these hypoxia-mediated miRNAs might provide a novel and effective therapeutic strategy for the prevention and/or treatment of solid tumors including PC.

In this study, we examined the effect of hypoxia on cell migration, invasion and angiogenesis, and expression of VEGF, IL-6, CSC signature genes, miR-21 and miR-210 in PC cells under hypoxic conditions. We also investigated the role of miR-21 in the regulation of VEGF, IL-6, the formation of pancreatospheres, and CSC signature genes in PC cells. Furthermore, we examined the effect of a novel synthetic derivative of curcumin (CDF) on cell survival, migration, invasion, angiogenesis, formation of pancreatospheres, and the expression of HIF-1α, VEGF, IL-6, CSC signature genes, and miRNAs in PC cells under hypoxic conditions. Finally, we examined the effect of CDF on miR-21, miR-210, HIF-1α, VEGF, and CSC signature markers *in vivo*.

## Materials and Methods

### Reagents and antibodies

A novel curcumin-derived analogue CDF was synthesized as described in our earlier publication [16]. Antibodies against CD44, EpCAM, and HIF-1α were obtained from Cell Signaling Technology (Beverly, MA). Antibodies against Ki-67 and VEGF were purchased from Santa Cruz (Santa Cruz, CA). Antibody against EZH2 was obtained from BD Biosciences (San Jose, California). Alexa Fluor-488 goat anti-mouse IgG for CD44 and EpCAM staining were obtained from Invitrogen. The miRNA reverse transcription (RT) primers, PCR probes, and anti-miR-21 inhibitor were obtained from Applied Biosystems (Carlsbad, CA). Crystal violet and Matrigel solution were obtained from Sigma Chemicals (St Louis, MO).

### Cell culture

Human pancreatic cancer (PC) cell lines AsPC-1 and MiaPaCa-2 cells were maintained at the standard culture or normoxic conditions (21% O2 and 5% CO2, 37°C), as described previously [17]. Hypoxic (1% O_2_) and 5% CO_2_ conditions were generated by the control of input flow rates of nitrogen and carbon dioxide, respectively. All the cell lines were maintained in 10% FBS-DMEM medium at a standard cell culture condition. All the cell lines have been authenticated by Applied Genomics Technology Center at Wayne State University on March 13, 2009 and these authenticated cells were frozen for subsequent use. The method used for testing was short tandem repeat profiling using the PowerPlex 16 System from Promega. Gemcitabine

### Cell survival assay

MTT assay was conducted to investigate the effect of CDF on cell survival in human PC cells (AsPC-1 and MiaPaCa-2 cells) under hypoxic conditions. 3,000 cells were seeded each well of the 96-well plates and incubated at standard culture conditions (21% O_2_ and 5% CO_2_) overnight. The cells were then treated with different concentrations of CDF (0–0.5 µM) and incubated for 8 h under hypoxic conditions (1% O_2_) followed by 16 h of normoxic conditions (21% O_2_) each day. After 3 days of treatment, the cells were harvested for the standard MTT assay, as described in our previous publications [18], [19].

### Clonogenic assay

Clonogenic assay was conducted to examine the effect of CDF on cell growth and proliferation of PC cells under hypoxic conditions, as described previously [18]. 5×10^4^ cells were plated in a 6-well plate. After 3 days of exposure to 0.5 µM of CDF (8 h of hypoxic conditions and 16 h of normoxic conditions each day), the cells were trypsinized, and 1,000 single viable cells were seeded in 100-mm Petri dishes. The cells were then incubated for 10 to 12 days at 37°C in a 5% CO_2_/5% O_2_/90% N_2_ incubator. Colonies were stained with 2% crystal violet, washed with water, and counted.

### Invasion assay

The *in vitro* invasion assay of PC cells was conducted under hypoxic conditions by using Costar Transwell 24-well-plates with polycarbonate membrane (Corning Incorporated, Corning, NY), as described previously [19]. Briefly, 4×10^4^ of human PC cells (AsPC-1 and MiaPaCa-2) exposed to 3 days of incubation under normoxic or hypoxic conditions, respectively, were seeded into each well of the Matrigel pre-coated Transwell plates in FBS-free culture media. The bottom wells of the system were filled with 10% FBS complete medium. After 20 h of incubation either in the absence or presence of CDF (0.5 µM), the invaded cancer cells were stained with 4 µg/mL of calcein-AM (Invitrogen) in PBS solution at 37°C for 1 h, following the manufacturer's manual. The photographs were taken using a fluorescent microscope (Nikon ECLIPSE TE2000-U) linked to a computer.

### Wound healing assay

To examine the effect of CDF on cell migration of PC cells under hypoxic conditions, we conducted wound healing assay, as described previously [17]. Briefly, when AsPC-1 cells reached 90–95% confluent, the wound was generated by scratching the surface of the plates with a 10–200 µL of pipette tip. The cells were then incubated in the absence and presence of CDF (0.5 µM) and were incubated under hypoxic conditions for 4 h, followed by 16 h of normoxic conditions, and then photographed using a Nikon Eclipse TS100 microscope.

### Tube forming assay

To examine the effect of CDF on angiogenesis *in vitro* using vascular endothelial cells under hypoxic conditions, we conducted tube formation assay, as described previously [20]. Briefly, 3×10^4^ rabbit vascular endothelial cells were seeded in each well of the Matrigel-pre-coated 96-well plate in 100 µL of 10% FBS-DMEM medium, and exposed to normoxic or hypoxic conditions for 4 h of incubation at 37°C, followed by 16 h of normoxic conditions. The photograph was taken at 4 h and 20 h, respectively.

### Cytokines VEGF and IL-6 assay

ELISA assay was conducted to assess the effect of CDF on hypoxia-induced cytokine productions of VEGF and IL-6 by human PC cells. The culture media from the cells under hypoxic or normoxic conditions, respectively, for 16 h were harvested for the measurement of VEGF and IL-6 by using ELISA assay kits (R&D Systems), following the manufacturer's manual.

### Sphere formation assay

The sphere formation assay was conducted to assess the effect of CDF on the CSC self-renewal capacity of PC cells under hypoxic conditions, as described previously [19]. Briefly, 1,000 cells/well of single cell suspensions were seeded in ultra low adherent wells of the 6-well plates (Corning, Lowell, MA) in sphere formation medium (1∶1 DMEM/F-12 medium supplemented with B-27 and N-2; Invitrogen), and exposed to hypoxic conditions every other day. After 7 days, the spheres with the diameter greater than 50 µmeters termed as “pancreatospheres” were collected by centrifugation (300× g for 5 min), and counted.

### Immunostaining assay and confocal microscopy

10,000 of single cell suspensions of PC cells per well were seeded and incubated for 24 h using ultra low adherent wells of 6-well plate (Corning, Lowell, MA) in sphere formation medium followed by culturing under hypoxic conditions every other day, as described above. After 7 days of CDF treatment, the pancreatospheres were collected by centrifugation (300× g for 5 min), wash with 1×PBS, and fixed with 3.7% parformaldehyde for 10 min at room temperature. Monoclonal CD44 and EpCAM antibodies were used for immunostaining assay, following the manufacturer's protocol, as described previously [17], [21]. The CD44 or EpCAM-labeled pancreatospheres were photographed using a confocal imaging microscope (Leica TCS SP5) with 200× magnification in MIRL Core facility, Wayne State University School of Medicine.

### Transfection of anti-miR-21 siRNA

2×10^5^ cells/well (parental MiaPaCa-2 cells) or 10,000 cells (MiaPaCa-2 sphere cells) per well were seeded in six-well plates and transfected with anti-miR-21 siRNA or negative control siRNA (Ambion, Austin, TX) at a final concentration of 20 nM using DharmaFECT transfection reagent (Dharmacon), following the manufacturer's protocol, as described previously [17].

### Real-time reverse transcriptase-polymerase chain reaction (RT-PCR) of mRNAs and miRNAs

To determine the relative mRNA levels, two micrograms of total RNAs extracted from each sample were used for RT reaction in 20 µL of reaction volume using a reverse transcription system (Invitrogen) according to the manufacturer's instruction. SYBR Green PCR Assay kit (Applied Biosystems, Carlsbad, CA) was used for real time PCR reaction, using AB StepOnePlus Real-Time PCR System (Applied Biosystems), following the manufacturer's protocol. Sequences of PCR primers were described previously [19]. The data were analyzed using C_t_ method and were normalized to GAPDH expression in each sample. To determine the expression of miRNAs in the cells, the TaqMan MicroRNA Assay kit (Applied Biosystems) was used following manufacturer's protocol. Five ng of total RNA was reverse transcribed as described earlier [19]. Real-time PCR reactions were then carried out in a total volume of 10 µL reaction mixture using TaqMan PCR solution as described earlier [17]. The data were analyzed using C_t_ method and were normalized by RNU48 expression in each sample.

### Animal experiments

The protocol of animal experiment was approved by the Animal Investigation Committee, Wayne State University, Detroit, MI. Female CB17 severe combined immuno-deficient (SCID) mice at the age of 4 weeks old were purchased from Taconic Farms (Germantown, NY) and were fed Lab Diet 5021 (Purina Mills, Inc., Richmond, IN). 10×10^6^ MiaPaCa-2 cells were orthotopically implanted into the pancreas of the mice as described in our earlier publication [17], and thus the anti-tumor activity data is not presented here. Once the mice developed palpable tumors, the animals were randomly divided into two groups: (1) untreated control; (2) CDF (5 mg/mouse/day), intragastric once daily for 12 days. Tumor measurements and changes in weight were performed. The tumor tissue from all groups of the animals were removed, rapidly frozen in liquid nitrogen, and stored at −80°C for later use for RNA and histology study as presented in this manuscript.

### Establishing MiaPaC-2 tumor sphere cells

In order to examine the effect of CDF or anti-miR-21 on VEGF, IL-6, CSC signatures in CSC-like subpopulation of tumor cells, we developed MiaPaCa-2 tumor sphere cells in mouse xenograft model, as described previously [22]. Briefly, about 5,000 pancreatospheres of MiaPaCa-2 cells were implanted in the xenograft mouse model (4 weeks of age) for enriching the cancer stem cell (CSC) population. The tumor was removed and the cells were cultured in the sphere formation medium, as described above, to develop the secondary pancreatospheres, which is referred as “MiaPaCa-2 tumor sphere cells”. MiaPaCa-2 tumor sphere cells were also characterized in our earlier publications [17], [22].

### Immunohistochemistry

Formalin-fixed and paraffin-embedded tumor tissue sections were evaluated by immunohistochemistry in the core facility of the Department of Pathology, Karmanos Cancer Institute, Detroit, MI, as described previously [17]. Briefly, 5–15 µm thick tissue sections were cut and mounted onto gelatin-coated histological slides. Deparaffinizations of the slides were performed by using xylene solution, followed by washing with different concentrations of alcohol and deionized water. Immunohistochemical staining was performed using Ki-67 (1∶400), HIF-1α (1∶100), VEGF (1∶100), EpCAM (1∶50), and EZH2 (1∶50) primary antibodies with appropriate dilutions and using normal host serum for negative controls followed by staining with appropriate horseradish peroxidase-conjugated secondary antibodies, following the manufacturers' instruction. The slides were developed in a solution of diaminobenzidine and counterstained with a weak solution of hematoxylin by using ABC Reagent kit (Vector Labs), followed by nuclear staining by using AEC staining kit (Vector Labs). The stained slides were dehydrated and mounted in Permount solution and examined by a licensed pathologist. The photographs were taken using a Nikon ESLIPSE E800 with 20× magnification. The immunohistochemical staining received an overall score based on the intensity of staining (no staining as zero; weak as 1+; moderate as 2+; strong as 3+) added together with the percent positive cells (no staining as zero; 25% as 1+; 25–50% as 2+ and greater than 50% as 3+).

### Statistical analysis

The mean and standard deviation (SD) of data in this study were prepared using GraPad Prism software (version 4.03). Comparisons of treatment outcome were tested for significant difference by the paired *t* test or ANOVA analysis. Statistical significance was assumed at a *p* value of less than 0.05.

## Results

### CDF increased cell survival and clonogenicity of PC cells under hypoxic conditions

**Figure 1:**
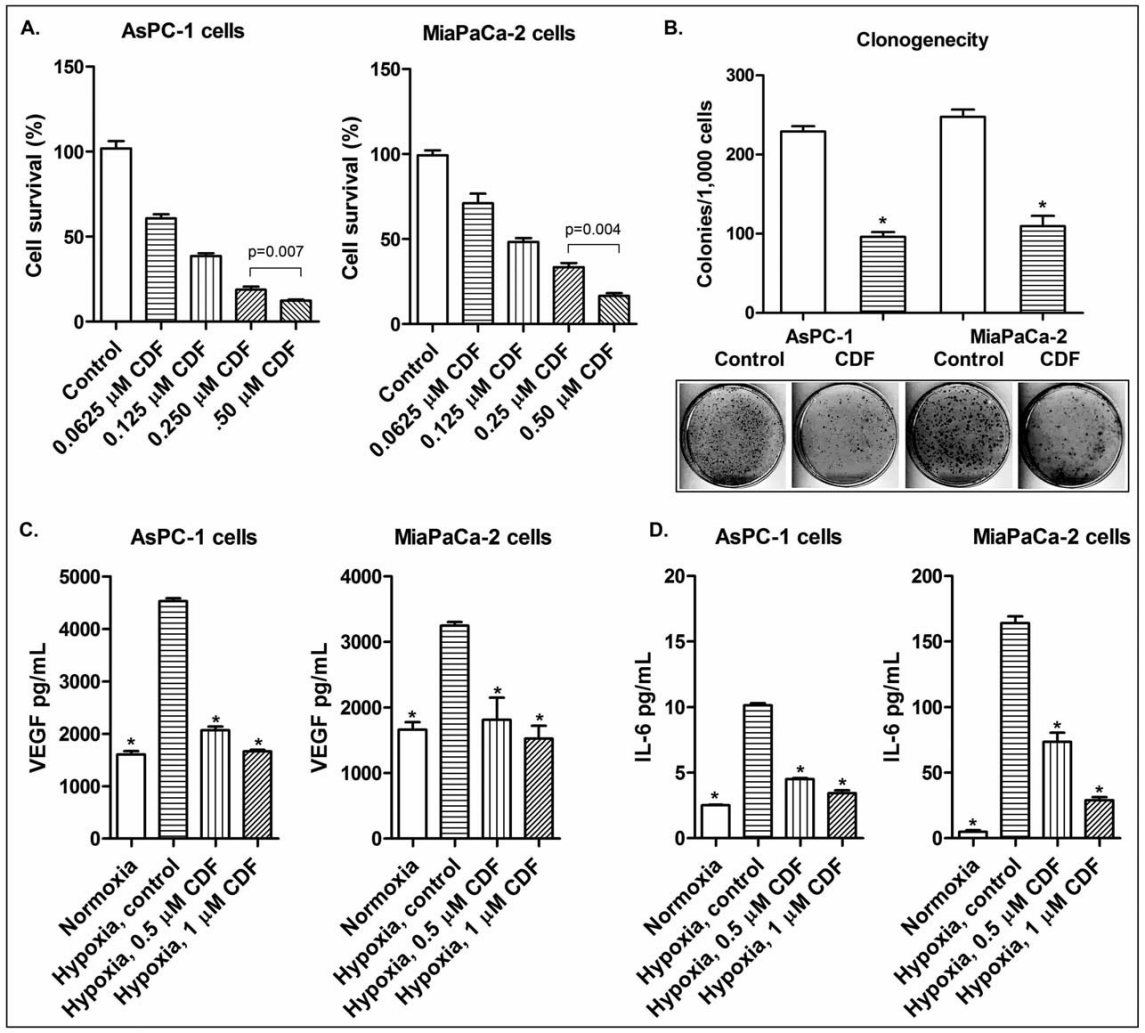
Effect of CDF on cell growth, clonogenicity, and on the expression of VEGF and IL-6 in human PC cells under hypoxic conditions. The panels A, B, C, and D represent cell growth, clonogenicity, VEGF, and IL-6, respectively. The conditioned media were collected from cells grown under normoxic and hypoxic conditions, as described under the methods section. The measurements of VEGF and IL-6 were conducted by ELISA. The bars in the charts indicate standard deviation (SD) of at least n = 3. * indicates p<0.05.

In order to investigate the effect of CDF on cell survival in human PC cells under hypoxic conditions, MTT assay was conducted using human PC cells (AsPC-1 and MiaPaCa-2 cells). The results indicate that CDF remarkably inhibited cell survival of AsPC-1 and MiaPaCa-2 cells in a dose-dependent manner (Figure 1A). Clonogenic assay was conducted to examine the effect of CDF (0.5 µmol/L) on cell growth and proliferation of PC cells under hypoxic conditions. The results showed that CDF treatment decreased clonogenicity of AsPC-1 and MiaPaCa-2 cells under hypoxic conditions (Figure 1B). These finding suggest that CDF inhibits cell survival and clonogenic growth of PC cells under hypoxic conditions.

### Effect of CDF on the productions of VEGF and IL-6 cytokines in PC cells under hypoxic conditions

We also examined the effect of CDF on hypoxia-induced VEGF and IL-6 productions by PC cells using ELISA assay. The results show that the AsPC-1 and MiaPaCa-2 cells incubated at hypoxic conditions increased the productions of VEGF and IL-6, compared to the cells incubated at normoxic conditions (Figure 1C). CDF treatment remarkably decreased the production of hypoxia-induced VEGF and IL-6 in PC cells (Figure 1C), suggesting an inhibitory role of CDF in the productions of hypoxia-induced VEGF and IL-6 in human pancreatic cancer cells.

### Effect of CDF on angiogenesis *in vitro* in vascular endothelial cells under hypoxic conditions

**Figure 2:**
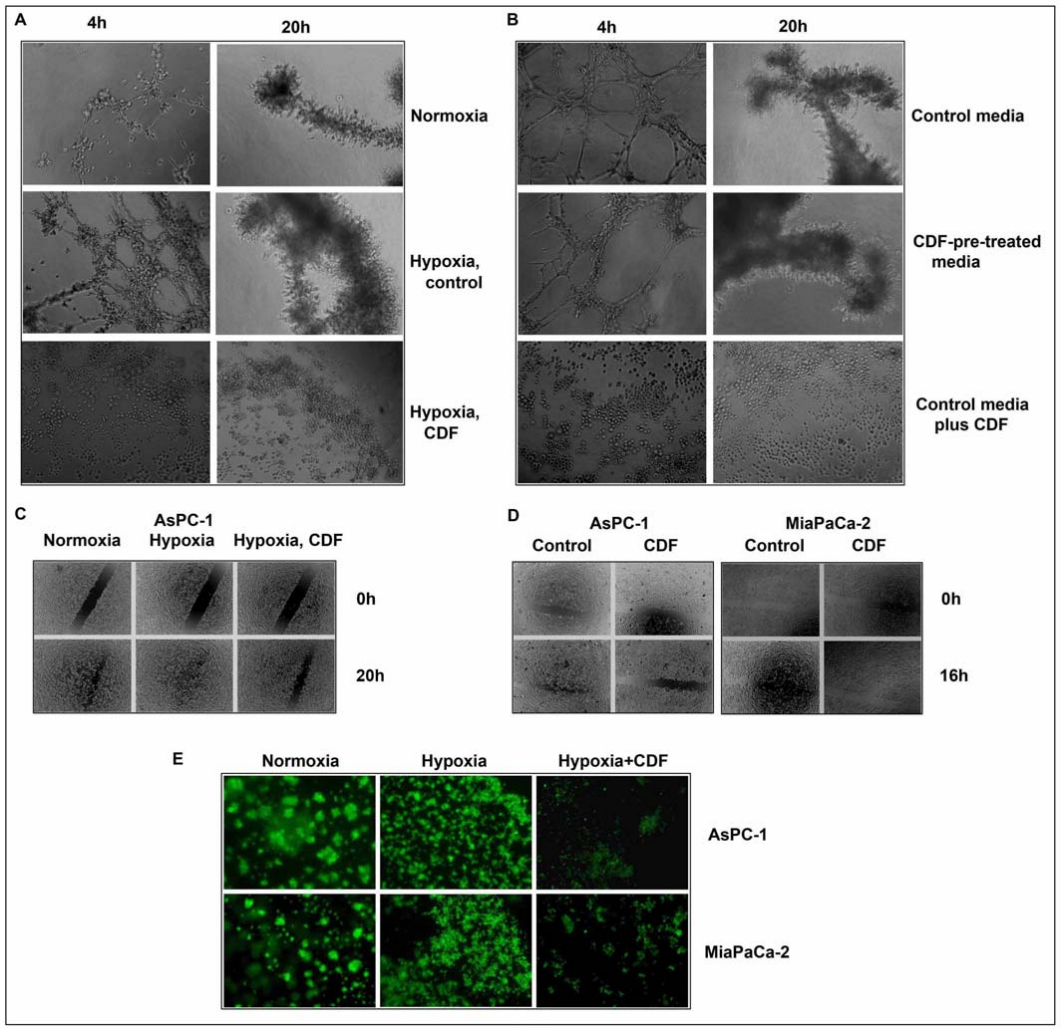
Effect of CDF on angiogenesis, cell migration, and invasion in PC cells under hypoxic conditions. The panels A&B, C&D, and E represent angiogenesis, cell migration, and invasion, respectively. As described in the Methods section, angiogenesis *in vitro* was evaluated by the tube formation assay using endothelial cells; cell migration was evaluated by wound healing assay; invasion was evaluated by chamber invasion assay.

In order to examine the effect of CDF on angiogenesis *in vitro* under hypoxic conditions, we conducted tube formation assay using vascular endothelial cells. The results show that hypoxic conditions significantly increased the tube formation of vascular endothelial cells at 4 h and 20 h of incubations, respectively, compared to normoxic conditions (p = 0.0016 and p = 0.016, respectively; n = 3). CDF treatment significantly inhibited the hypoxia-induced tube formation of vascular endothelial cells (p = 0.004; n = 3) (Figure 2A). To assess whether or not CDF-mediated molecules or CDF itself contributes to the inhibition of tube formation, we collected non-CDF-treated (control) and CDF-treated conditioned media from cancer cells and conducted the tube formation assay under normoxic conditions. As shown in Figure 2B, the vascular endothelial cells incubated with control condition media had increased tube formation at 4 h and 20 h, compared to the cells incubated with CDF-pre-treated conditioned media (p = 0.029 and p = 0.011; n = 3). Addition of CDF to the control conditioned media significantly inhibited the tube formation, compared to the cells incubated at both the control conditioned media and CDF-pre-treated conditioned media (p = 0.0001; n = 3) (Figure 2B). These data suggest that CDF itself contributes to the inhibition of the tube formation of vascular endothelial cells.

### Effect of CDF on cell migration and invasion in vitro in PC cells under hypoxic conditions

The wound healing assay was conducted to examine the effect of CDF on cell migration of PC cells under hypoxic conditions. As shown in Figure 2C, the hypoxia-exposed AsPC-1 cells had increased the wound healing capacity, compared to the cells cultured under normoxia (p = 0.0001; n = 3) (Figure 2C). CDF treatment inhibited the wound healing capacity in the cancer cells under hypoxic conditions (p<0.05; n = 3) (Figure 2C and D). To examine the effect of CDF on invasion of PC cells under hypoxic conditions, we conducted *in vitro* invasion assay. As shown in Figure 2E, both AsPC-1 and MiaPaCa-2 cells exposed to hypoxic conditions had increased the capacity of invasion, compared to those cells exposed to normoxic conditions. CDF treatment inhibited the capacity of hypoxia-induced invasion of PC cells. These data suggest that CDF can inhibit hypoxia-induced cell migration and invasion of human PC cells.

### Effect of CDF on the gene expression of CSC markers in PC cells under hypoxic conditions

**Figure 3:**
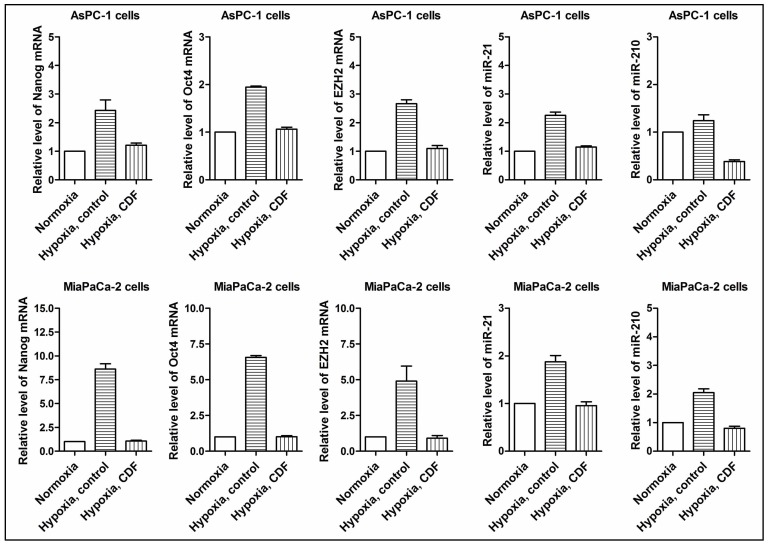
Effect of CDF on the expression of Nanog, Oct4, EZH2, miR-21, and miR-210 in PC cells under hypoxic conditions. Real time RT-PCR was employed as described under the Methods section. The bars in the charts indicate SD of n = 3. * indicates p<0.05.

The real time RT-PCR assay was conducted to examine the effect of CDF on cancer stem cell (CSC) signature genes in AsPC-1 and MiaPaCa-2 cells under hypoxic conditions. As shown in Figure 3, hypoxia induced the relative mRNA levels of Nanog, Oct4, and EZH2 in AsPC-1 and MiaPaCa-2 cells whereas CDF decreased the levels of Nanog, Oct4, and EZH2 mRNAs in PC cells under hypoxic conditions.

### Effect of CDF on the microRNA (miRNA) expression of miR-21 and miR-210 in PC cells under hypoxic conditions

In order to examine the effect of CDF on miRNA expression in PC cells under hypoxic conditions, we measured the relative levels of miR-21 and miR-210 under hypoxic conditions by real-time RT-PCR. As shown in Figure 3, hypoxia induced the relative miRNA levels of miR-21 and miR-210 in AsPC-1 and MiaPaCa-2 cells whereas CDF decreased the levels of miR-21 and miR-210 in PC cells under hypoxic conditions, suggesting that CDF is a potent agent in attenuating hypoxia-induced expression of miR-21 and miR-210 in human PC cells.

### Effect of CDF or anti-miR-21 on the CSC self-renewal capacity in human PC cells under hypoxic conditions

**Figure 4:**
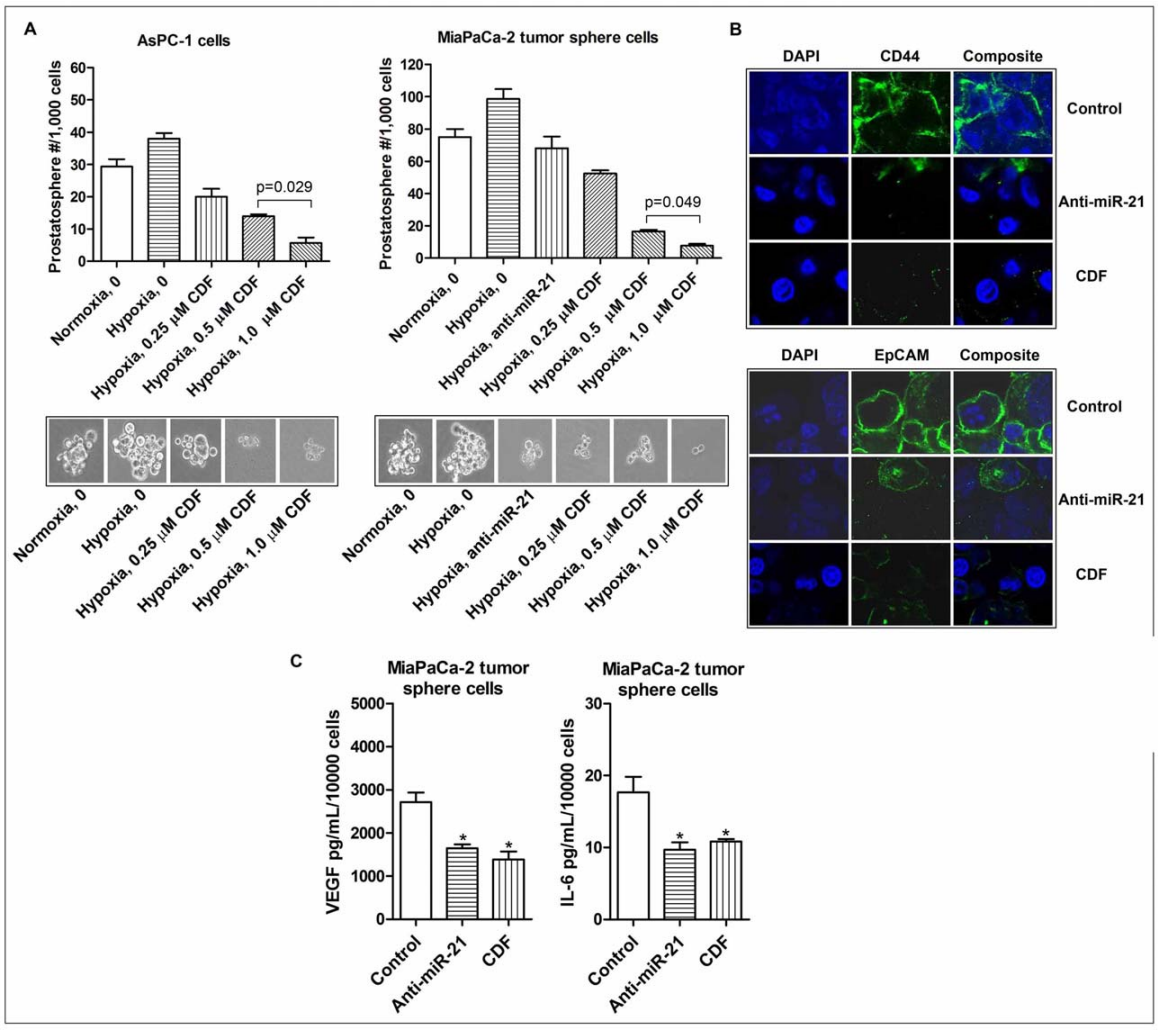
Effect of CDF and anti-miR-21 on the formation of pancreatospheres, the expression of CD44 and EpCAM, and the production of VEGF and IL-6 in the CSC-like sphere cells of PC cells under hypoxic conditions. The panels A, B, and C represent the formation of pancreatospheres, the expression of CD44 and EpCAM, and the production of VEGF and IL-6, respectively. The sphere formation assay was conducted to examine the self-renewal capacity of CSCs in PC cells, as described under the Methods section. Confocal imaging microscopy (200× magnification) was conducted to measure CSC cell surface markers CD44 and EpCAM in the MiaPaCa-2 tumor sphere cells, as described under the Methods section. The bars in the charts indicate SD of n = 3. * indicates p<0.05.

In order to assess the effect of CDF or anti-miR-21 on the CSC self-renewal capacity of human PC cells under hypoxic conditions, we conducted the sphere formation assay using AsPC-1 and MiaPaCa-2 cells. The results show that anti-miR-21 decreased the formation of pancreatospheres in MiaPaCa-2 cells (Figure 4A). These data suggest that miR-21 may play an important role in the regulation of the self-renewal capacity of CSC-like PC cells. Similarly, CDF treatment decreased the formation of pancreatospheres in AsPC-1 and MiaPaCa-2 cells under hypoxic conditions (Figure 4A).

### Effect of CDF or anti-miR-21 on the expression of CSC cell surface marker proteins CD44 and EpCAM in PC cells-derived sphere cells under hypoxic conditions

To examine whether or not CDF or anti-miR-21 inhibits the expression of CSC cell surface markers CD44 and EpCAM in CSC-like cells derived from PC cells, we conducted confocal imaging microscopy for assessing the expression of CD44 and EpCAM in the MiaPaCa-2 cells initiated tumor-derived sphere cells. The results show that anti-miR-21 decreased the expression of CD44 and EpCAM in MiaPaCa-2 tumor sphere cells under hypoxic conditions, consistent with the results from CDF treatment (Figure 4B). These data suggest that CDF down-regulates the formation of MiaPaCa-2 tumor sphere cells consistent with down-regulation of CD44 and EpCAM expression, which is in part mediated through decreased expression of miR-21.

### Effect of CDF or anti-miR-21 on VEGF and IL-6 production in the MiaPaCa-2 tumor sphere cells under hypoxic conditions

As shown in Figure 4C, MiaPaCa-2 tumor sphere cells produced relatively larger amount of VEGF under hypoxic conditions, compared to its parental MiaPaCa-2 cells (2174 pg/mL/10^4^ MiaPaCa-2 tumor sphere cells vs 3248 pg/mL/10^6^ MiaPaCa-2 cells; Figure 1C and 4C), suggesting that MiaPaCa-2 tumor spheres may promote angiogenesis by up-regulating the production of VEGF. We also found that CDF treatment decreased hypoxia-induced VEGF production in MiaPaCa-2 tumor sphere cells. Similarly, MiaPaCa-2 sphere cells produced relatively higher amount of hypoxia-induced IL-6, compared to its parental cells (18 pg/mL/10^4^ MiaPaCa-2 sphere cells vs 164 pg/mL/10^6^ parental MiaPaCa-2 cells; Figure 1D and 4C). CDF treatment or deficiency of miR-21 by anti-miR-21 transfection decreased the production of hypoxia-induced IL-6 in the CSC-like sphere cells (Figure 4C).

### Effect of CDF or miR-21 deficiency on the gene expressions of HIF-1α, VEGF, IL-6, CD44, EpCAM, and EMT phenotype markers in MiaPaCa-2 tumor sphere cells under hypoxic conditions

**Figure 5:**
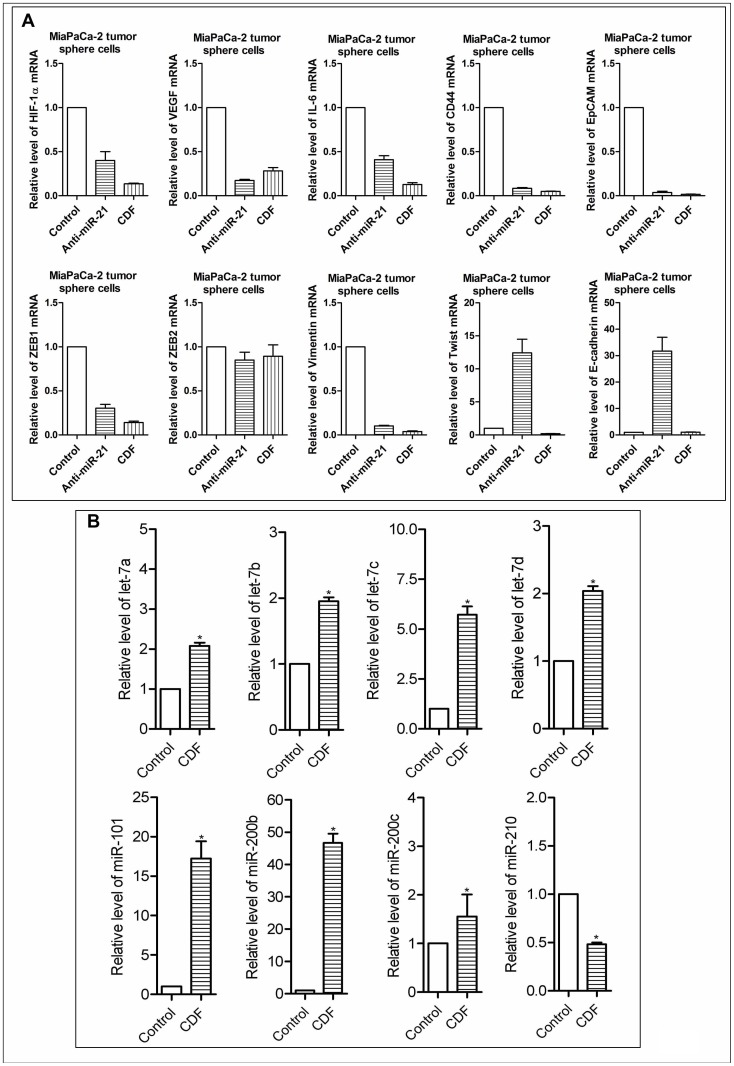
Effect of CDF and/or anti-miR-21 on the mRNA and miRNA expressions in the CSC-like sphere cells of PC cells under hypoxic conditions. The panels A and B represent the mRNAs and miRNAs, respectively. The sphere cells were transfected with anit-miR-21 by using the transfection reagent, following the manufacturer's manual. Real time RT-PCR was employed as described under the Methods section. The bars in the charts indicate SD of n = 3. * indicates p<0.05.

As shown in Figure 5A, CDF treatment decreased the relative mRNA levels of HIF-1α, VEGF, IL-6, CD44, and EpCAM in MiaPaCa-2 tumor sphere cells under hypoxic conditions. Similarly, down-regulation of miR-21 by anti-miR-21 transfection decreased the expression of these genes in MiaPaCa-2 tumor sphere cells under hypoxic conditions. We also examined whether or not CDF or miR-21 deficiency regulates the gene expression of EMT markers in MiaPaCa-2 tumor sphere cells under hypoxic conditions. We found that inactivation of miR-21 expression resulted in a significant decrease in the relative mRNA levels of EMT mesenchymal markers ZEB1 and Vimentin, and increased the relative mRNA level of epithelial marker E-cadherin in the tumor sphere cells under hypoxic conditions (Figure 5A). Similarly, CDF decreased the mRNA levels of ZEB1, Vimentin, and Twist in the tumor sphere cells under hypoxic conditions (Figure 5A).

### Effect of CDF on the miRNA expression in MiaPaCa-2 tumor sphere cells under hypoxic conditions

Recent experimental evidence has shown that hypoxia could regulate the expression of a number of miRNAs, including anti-oncogenic (let-7 and miR-101), and pro-oncogenic (miR-21 and miR-210) miRNAs [10], [12], [13], [15], [23], [24]. In order to further examine the effect of CDF on the expression of these miRNAs, we measured the levels of these miRNA in MiaPaCa-2 tumor sphere cells under hypoxic conditions by real-time RT-PCR. The results show that CDF increased the relative miRNA levels of let-7c,d, miR-101, and miR-200b and decreased the relative levels of miR-210 in MiaPaCa-2 tumor sphere cells under hypoxic conditions (Figure 5B). These data suggest that CDF could deregulate hypoxia-associated miRNAs in CSC-like cells of PC.

### Effect of CDF on tumor growth and the expression of Ki-67, EZH2, HIF-1α, VEGF, EpCAM, miR-21, and miR-210 *in vivo*

**Figure 6:**
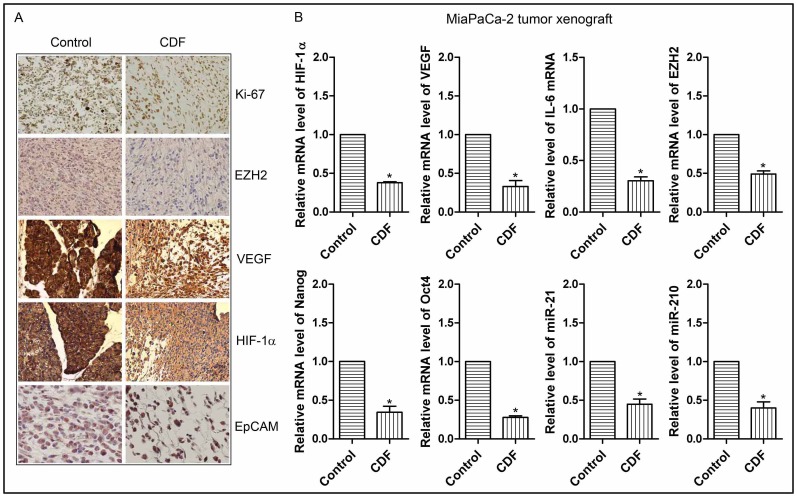
CDF decreased the expression of cell proliferation marker Ki-67, and other markers of tumor aggressiveness such as HIF-1α, VEGF, EZH2, EpCAM as well as the expression of HIF-1α, VEGF, IL-6, EZH2, Nanog, Oct4 mRNAs, miR-21, and miR-210 in pancreatic tumor tissues (orthotopic tumors induced by human MiaPaCa-2 cells). The panels A and B represent immunohistochemistry assessed by immunohistochemistry (20× magnification), and mRNAs/miRNAs assessed by real time RT-PCR, respectively. The bars in the charts indicate SD of n = 3. * indicates p<0.05.

We conducted the animal experiment in orthotopic mouse model by the injecting MiaPaCa-2 cells into the pancreas for the development of tumor. In this model, we have previously reported that CDF treatment could significantly decrease the pancreatic tumors, compared to the control animals without CDF treatment [17]. We have used the tumor tissues from control and CDF-treated animals and here we show that CDF treatment led to decreased expression of Ki-67, EZH2, HIF-1α, VEGF, and EpCAM in pancreatic tumor remnants as assessed by immunohistochemistry (Figure 6A). We evaluated the staining and given an overall score (staining intensity was added with % positive cells) as documented under methods section. Specifically, for Ki-67, the control and CDF was 5 vs 0; EZH2, 5 vs 2; VEGF, 6 vs 4; HIF-1alpha, 6 vs 4; EpCAM, 5 vs 4, respectively. We also examined the effect of CDF treatment on the expression of miR-21 and miR-210, and the mRNA expression of HIF-1α, VEGF, and CSC signature markers in the tumor tissues. The results show that CDF treatment significantly decreased the relative levels of miR-21 and miR-210, and mRNA expression of HIF-1α, VEGF, IL-6, EZH2, Nanog, and Oct4 the tumor tissues (Figure 6B), suggesting that CDF in a tumor model *in vivo* could inhibit the expression of miRNAs and mRNAs that are associated with CSCs and deregulated under hypoxic conditions *in vitro*.

## Discussion

The data from a number of epidemiological and clinical studies have suggested that hypoxia and hypoxia-induced signaling pathways are highly associated with poor clinical outcome of patients diagnosed with many solid tumors including PC. Moreover, the evidence from many experimental studies suggests that hypoxia enhances cell migration, invasion, and angiogenesis, which can contribute to tumor aggressiveness. Here, we confirm that hypoxia induces cell migration, and invasion, and angiogenesis *in vitro*, and consistent with increased production of VEGF in PC cells. We also found that hypoxia induces the formation of pancreatospheres in PC cells, consistent with increased expression of CSC signature markers, such as EZH2, Oct4, CD44, and EpCAM in PC cells. It has been documented that hypoxia is clearly involved in the maintenance of cancer stem cell (CSC) phenotype which function through HIF proteins and its downstream gene targets such Oct4, and Notch-1 [7], [25], [26]. These additional data suggest that hypoxia may have an important role in the regulation of CSC characteristics, resulting in the promotion of tumor aggressiveness.

Recent years have witnessed tremendous advances in miRNA research, and miRNAs have been widely recognized to play a critical role in tumorigenesis mediated through the regulation of mRNA degradation or inhibition of protein translation through its binding to the 3′ un-translated region of target genes. It has been widely accepted that miR-21 might function as a pro-oncogenic molecule, which promote the development and progression of tumors. The clinical studies have also shown that higher expression of miR-21 is associated with poorer clinical outcome in many solid tumors including PC [27], [28]. It has been reported by our laboratory that increased expression of miR-21 leads to decreased expression of PTEN, a known tumor suppressor, which inhibits PI3K/Akt signaling pathway in cancers [17], [18]. Higher expression of miR-21 has been found in several cancer cell lines under hypoxic conditions [11], [24]. It has been also noted that forced over-expression of miR-21 in human prostate cancer DU145 cells led to increased expression of HIF-1α and VEGF mediated through Akt and ERK pathways, consistent with an increase in tumor angiogenesis [29]. Moreover, decreased expression of miR-21 by anti-miR-21 transfection led to increased expression of PTEN, an endogenous inhibitor of Akt and ERK pathway, and inhibited tumor angiogenesis through the down-regulation of HIF-1α and VEGF in cancer cells [29]. Thus, HIF-1α appears to be a key downstream target of miR-21 in the regulation of tumor angiogenesis, even though a putative binding site of miR-21 to 3′ UTR of either HIF-1α or VEGF has been not identified. It has also been shown that forced over-expression of miR-21 could promote the survival of bone marrow mesenchymal stem cells under hypoxic conditions, and down-regulation of miR-21 by its siRNA inhibitor led to increased apoptotic potential of mesenchymal stem cells [30]. Here, we have shown that hypoxia resulted in increased expression of miR-21 in PC cells, and also associated with increased expression of VEGF, and the self-renewal capacity of CSCs. The loss of miR-21 expression by anti-miR-21 transfection resulted in decreased expression of VEGF, CD44, EpCAM, and decreased CSC self-renewal capacity in PC cells derived CSC-like sphere-forming cells under hypoxic conditions, which clearly suggest that hypoxia-induced miR-21 expression may play a pivotal role within the tumor microenvironment, contributing to the promotion of tumor aggressiveness. However, further in-depth investigation is required to elucidate the exact role of hypoxia-induced miR-21 in the phenotype and function of CSCs within the tumor microenvironment.

A large body of experimental evidence has shown that miR-210 is a hypoxia-induced miRNA in a variety of cells including cancer cells [7]. The higher expression of miR-210 has been reported to be highly associated with poor clinical prognosis in PC and other cancers [31]. The data from several experimental studies have indicated that hypoxia-induced higher expression of miR-210 is associated with hypoxia-induced downstream targets VEGF and carbonic anhydrase 9 (CAIX) in cancer cells by a HIF-1α-dependent mechanism [15], [32], which suggests that miR-210 may have a regulatory role in the promotion of tumor angiogenesis [11]–[13], [23], [33], [34]. In this study, we found that hypoxia led to an increase in the expression of miR-210 in PC cells and CSC-like sphere cells. These findings suggest that hypoxia-induced miR-210 may play an important role in the tumor microenvironment, which further suggest that targeting miR-210 would provide a novel therapeutic strategy such as the usefulness of CDF for the treatment of human malignancies.

IL-6, a known inflammatory cytokine, has been reported to play a critical role in tumorigenesis. The clinical data have shown a strong association between higher expression of IL-6 with poor clinical outcome of patients in a variety of tumors including PC [35]. Several experimental studies have demonstrated that IL-6 promotes tumorigenesis, angiogenesis, metastasis, and treatment resistance [36]–[39]. Moreover, the early evidence suggests that IL-6 may have an important role in the CSC phenotype and function [40], similar to the other recent findings documenting that lung CSC-like cells have a high level of expression of IL-6/IL-6R [38]. Additionally, IL-6 has been shown to enhance tumorigenicity in glioblastoma, consistent with an increase in the CSC self-renewal capacity [41], [42]. Here, we show for the first time, that the CSC-like sphere cells of PC cells produce a significant amount of IL-6, compared to its parental non-sphere cancer cells under hypoxic conditions. These findings clearly provide evidence to support that IL-6 may function as a direct mediator of the CSC's self-renewal capacity. However, the exact role of IL-6 in the regulation of CSC characteristics requires further in-depth investigations.

Curcumin (diferuloylmethane) is a bioactive natural compound [43], [44], and a large body of experimental studies have shown that curcumin can inhibit cell growth, cell invasion, tumor growth, and induce apoptotic cells death, which is consistent with deregulation of multiple cellular signaling pathways such as NF-κB, Notch, Akt/mTOR, Wnt, HIF-α, and Hedgehog [16], [45]–[50]. However, due to poor bio-availability of curcumin in human limits its application in the clinical setting, which led to our recent development of a novel synthetic analog of curcumin referred to as Difluorinated-Curcumin (CDF) [16], which showed greater bioavailability in multiple tissues including pancreas. Our earlier studies have shown that CDF could function as a potent anti-tumor agent against human pancreatic tumor *in vitro* and *in vivo* by regulating miRNAs, CSC phenotype and function, and multiple cellular signaling pathways such as NF-κB, Akt, COX-2, Notch-1, and EZH2 [17]–[19]. Here, we show that CDF can inhibit cell survival, clonogenicity, migration/invasion, and angiogenesis, and the CSC self-renewal capacity in human PC cells *in vitro* under hypoxic conditions, consistent with the inhibition of miR-21, miR-210, HIF-1α, and CSC signature gene markers. More importantly, our previously published study using a mouse orthotopic model of human PC showed anti-tumor activity of CDF [17], [19], which was consistent with inhibition of HIF-1α, VEGF, CSC signatures, miR-21, and miR-210. Collectively, these findings have provided convincing evidence suggesting that CDF could function as a novel anti-tumor agent, mediated through the deregulation of multiple signaling pathways including hypoxia-induced CSC phenotype and function within the tumor microenvironment, which contributes to the reduction of tumor aggressiveness of PC.
